# Microbial and antimicrobial resistance signatures differentiate healthy and inflamed ileal pouches in patients with ulcerative colitis

**DOI:** 10.1093/ecco-jcc/jjag150

**Published:** 2026-11-07

**Authors:** Sebastian Mølvang Dall, Alberte Holm Møllekær, Sabrina Just Kousgaard, Sarah Søgaard Jakobsen, Lone Larsen, Bertil Sørensen, Ole Thorlacius-Ussing, Mads Albertsen

**Affiliations:** Center for Microbial Communities, Department of Chemistry & Bioscience, Aalborg University, Aalborg, Denmark; Center for Microbial Communities, Department of Chemistry & Bioscience, Aalborg University, Aalborg, Denmark; Department of Gastrointestinal Surgery, Aalborg University Hospital, Aalborg, Denmark; Department of Gastroenterology and Hepatology, Aalborg University Hospital, Aalborg, Denmark; Department of Gastrointestinal Surgery, Aalborg University Hospital, Aalborg, Denmark; Department of Gastroenterology and Hepatology, Aalborg University Hospital, Aalborg, Denmark; Center for Molecular Prediction of Inflammatory Bowel Disease, PREDICT, Department of Clinical Medicine, Aalborg University, Copenhagen, Denmark; Center for Microbial Communities, Department of Chemistry & Bioscience, Aalborg University, Aalborg, Denmark; Department of Gastrointestinal Surgery, Aalborg University Hospital, Aalborg, Denmark; Center for Microbial Communities, Department of Chemistry & Bioscience, Aalborg University, Aalborg, Denmark

**Keywords:** pouchitis, ileal pouch–anal anastomosis (IPAA), gut microbiome, ulcerative colitis, antimicrobial resistance, metagenomics, plasmids

## Abstract

**Background and Aims:**

Fecal microbiota transplantation (FMT) from healthy donors with a normally functioning colon has limited clinical effect in the treatment of chronic pouchitis. However, a normally functioning ileal pouch–anal anastomosis (IPAA) physiology differs from individuals with a healthy intact colon. We aimed to investigate the microbial characteristics of patients with a normally functioning IPAA (HealthyPouch) compared with chronic pouchitis (SickPouch), ulcerative colitis (UC), and a healthy intact colon microbiome (NormalGut). Furthermore, we investigated how repeated antibiotic use influences antimicrobial resistance on a bacterial genome level.

**Methods:**

Fecal samples from 190 participants were collected; HealthyPouch (*n* = 38), SickPouch (*n* = 37), UC (*n* = 23), and NormalGut (*n* = 92) and sequenced with Illumina and taxonomically profiled. Samples from HealthyPouch, SickPouch, and NormalGut were also long-read sequenced using Oxford Nanopore Technologies for reconstruction of 12 282 metagenome-assembled genomes (MAGs). DNA methylation motifs were used for plasmid–host association.

**Results:**

The HealthyPouch microbiome is distinct from both the UC, NormalGut, and SickPouch microbiome. *Escherichia coli* MAGs in the SickPouch (*n* = 21) microbiome had significantly higher co-occurring ciprofloxacin resistance mutations in the *gyrA* and *parC* genes, compared to both HealthyPouch (*n* = 26; BH adjusted *P* < .05) and NormalGut (*n* = 40; BH adjusted *P* < .05). Based on DNA methylation pattern, three plasmids were associated with *E. coli* carrying extended-spectrum beta lactamase genes.

**Conclusions:**

HealthyPouch microbiota differs from NormalGut microbiota, and could therefore be more appropriate for FMT treatment of chronic pouchitis. The association between *E. coli* and resistance gene-carrying plasmids underscores the importance of integrating plasmid analysis into metagenome-based approaches for personalized antibiotic treatment and resistance monitoring.

## Graphical abstract

**Graphical Abstract:**
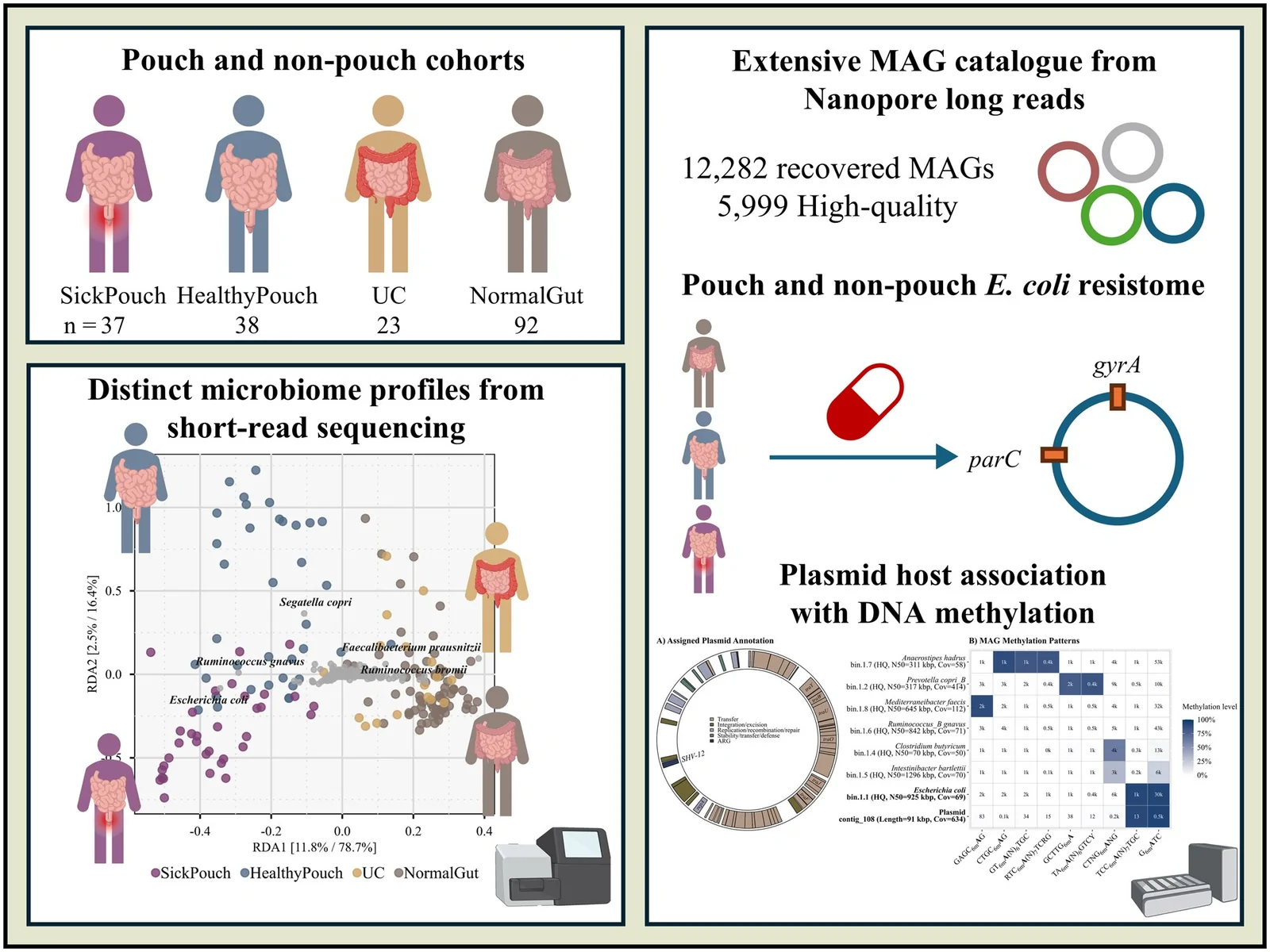

## 1. Introduction

Pouchitis is an inflammatory condition affecting the ileal pouch–anal anastomosis (IPAA), a surgical intervention for medically refractory ulcerative colitis (UC).^1^ Although medical therapy remains the primary treatment for UC, failure rates are substantial, with approximately 10% of patients eventually requiring a proctocolectomy.^2^ Pouchitis is the most prevalent long-term complication following IPAA surgery, with studies reporting variable incidence (14%-59%) due to differences in follow-up duration and diagnostic criteria.^1^ The pathogenesis of pouchitis is not fully elucidated but is believed to result from a combination of gut microbiota dysbiosis, mucosal immune dysregulation, and genetic predisposition.^3^ Notably, gut microbiome alterations are strongly implicated in disease progression.^4^ The first-line treatment for acute pouchitis consists of antibiotics, primarily metronidazole and/or ciprofloxacin.^5^^,^^6^ Approximately 20% of patients with pouchitis develop chronic inflammation, which may be either treatment-responsive, requiring continuous or recurrent antibiotic therapy, or treatment-refractory, failing to respond to standard antibiotic regimens.^7^ Many treatment options for antibiotic-refractory pouchitis have been introduced, such as immunomodulators, biologics, and small molecules, similar to the treatment of UC.^8^ In particular, treatment with vedolizumab has shown encouraging results in the EARNEST trial.^9^ The management of chronic pouchitis frequently involves prolonged or repeated antibiotic use; however, this high level of antibiotic exposure is associated with complications, including an increased risk of antimicrobial resistance within the gut microbiome.^1^ Patients with chronic pouchitis are at significant risk of pouch failure, with approximately 10% eventually requiring pouch removal and conversion to a permanent ileostomy.^10^

Fecal microbiota transplantation (FMT) is recognized as an effective treatment for recurrent *Clostridioides difficile* infection (rCDI) in the clinical setting and in several studies has also demonstrated efficacy in the management of UC.^11^^,^^12^ FMT has been proposed as a potential therapeutic approach for chronic pouchitis. However, three randomized controlled trials evaluating FMT in patients with chronic pouchitis reported no clinical efficacy compared to placebo.^13–15^ While one study observed slight microbiome alterations in the FMT group during treatment,^15^ these changes did not translate to significant change in clinical improvement compared to the placebo group. A previous study found that antibiotic resistance genes are highly prevalent among antibiotic-exposed patients with chronic pouchitis.^16^ Additionally, FMT has been shown to reduce the relative abundance of antibiotic resistance genes in patients with rCDI.^17^

As FMT from a healthy fecal donor has proven ineffective in treating chronic pouchitis, the present study aims to investigate the defining characteristics of a healthy pouch microbiome and assess the impact of high antibiotic consumption in patients with chronic pouchitis.

## 2. Methods

### 2.1. Study design

In this cohort study, we used fecal samples of four different participant groups: normally functioning IPAA (HealthyPouch), chronic pouchitis (SickPouch), ulcerative colitis (UC), and healthy controls (NormalGut), to assess the microbiome composition and antibiotic resistance genes.

Participants with chronic pouchitis (SickPouch) and participants with a normally functioning IPAA (HealthyPouch) were recruited between November 2017 and October 2022 at the Department of Gastrointestinal Surgery, Aalborg University Hospital in Aalborg, Denmark. Participants were recruited via telephone or during outpatient visits. All participants were 18 years of age or older and had had their IPAA for more than 1 year.

SickPouch participants were identified using patient records at the hospital. Eligible patients had pouchitis with a total Pouchitis Disease Activity Index (PDAI) score of ≥7 points, a clinical symptom sub-score of ≥3 points, and endoscopic and histologic sub-scores each ≥1 point.^18^ Chronic pouchitis was defined as three or more episodes of pouchitis diagnosed according to clinical symptoms and/or pouchitis with continued symptoms >4 weeks despite treatment with antibiotics, endoscopic signs of inflammation, and histologic inflammation of pouch biopsies with a PDAI score ≥7 within the last year. All had received one or more courses of antibiotic treatment within 12 months prior to inclusion in the study (median: 3 months), using either ciprofloxacin (a fluoroquinolone) and/or metronidazole (a nitroimidazole). Patients were excluded in the case of secondary pouchitis (such as Crohn’s disease), immunosuppression (diagnosed with human immunodeficiency virus, long-term treatment with prednisolone >3 months, received anti-TNF-alpha therapy, and/or chemotherapy), or treatment with probiotic supplements during the last 2 weeks before inclusion. Other exclusion criteria were a positive stool test for enteric bacterial pathogens including *Salmonella*, *Campylobacter*, *Yersinia*, *Shigella*, *Vibrio*, toxin producing *Clostridioides difficile*, and diarrhea-genic *Escherichia coli* including Shiga toxin-producing *E. coli* (STEC).

HealthyPouch participants with no symptoms of pouchitis after IPAA surgery for UC were identified using patient records at the hospital. Inclusion criteria were the absence of documented episodes of pouchitis, absence of symptoms of pouch dysfunction, a clinical PDAI score <3, and no use of antibiotics for pouchitis within the last 12 months prior to inclusion. Participants were excluded if their stool frequency exceeded eight stools per day.

NormalGut controls without disease in the colon or rectum were recruited between April 2019 and April 2024 from the Blood Bank at Aalborg University Hospital, Aalborg, Denmark, or through hospital advertisements. Stool frequency should range from one to three bowel movements per day, with a consistency of 3–4 on the Bristol Stool Chart. Participants were excluded if they had any diseases, were taking any medication (anti-contraceptive medicine was allowed), or had received antibiotic treatment 6 months prior to inclusion. Fecal donors in the fecal microbiota transplantation trials at Aalborg University Hospital were also included as NormalGut participants.^15^^,^^19^

UC participants were included at the Department of Medical Gastroenterology, Aalborg University Hospital, from January 2023 to January 2024. All 23 participants were 18 years of age or older and had been diagnosed with UC for more than 1 year. Eligible participants had UC in remission with the Simple Clinical Colitis Activity Index (SCCAI) < 5.^20^ Maintenance therapy was allowed with 13 participants receiving 5-aminosalicylic acid, two participants receiving biological agents, mainly infliximab, four patients received both 5-aminosalicylic acid and biological agents, and four participants received no maintenance therapy. Participants were excluded if they had received antibiotic treatment 6 months prior to inclusion or if their stool frequency exceeded six stools per day.

None of the participants in the HealthyPouch, UC, or NormalGut cohorts had received antibiotic treatment within 6 months prior to inclusion.

Each participant was asked to deliver a fecal sample when included in the study. The participants also completed a questionnaire to obtain information about daily stool frequency, use of any type of antibiotics within the last 6 months (including specific use of antibiotics for pouchitis [ciprofloxacin and/or metronidazole] and continuous use of antibiotics) and information about current pouch function for the pouch patients (using the clinical PDAI score, cPDAI) and activity score using SCCAI for patients with UC.

### 2.2. DNA extraction

Fecal sample DNA extraction was performed using the DNeasy® 96 PowerSoil® Pro QIAcube HT kit. Before extraction, 100 µL of fecal material was transferred to 1.2 mL 2D barcoded matrix tubes with lysing matrix E from MP biomedicals. Samples were organized in the 96-plate format. In total, 500 µL of CD1 was added to the samples before being subject to three bead-beating cycles performed in 2-min intervals at 1600 rpm using the FastPrep96™. Samples were kept on ice for 2 min during bead-beating cycles. Lysed material was centrifuged for 10 min at 3486 *g* in an Eppendorf 5810 benchtop centrifuge. After centrifugation, 300 µL of supernatant was transferred to a clean S-block containing 300 µL CD2 and 100 µL nuclease-free water (NFW) before repeating centrifugation as before. Subsequent steps followed the manufacturer’s protocol.^21^

### 2.3. Illumina library prep, sequencing, and pre-processing

Illumina metagenomic sequencing was performed for all collected fecal samples for all four study cohorts: SickPouch, HealthyPouch, UC, and NormalGut. Metagenome libraries were prepared with the Illumina DNA prep kit in 96-well plates using a miniaturized library volume protocol. Using the I.DOT One, a 5-µL reaction volume was created by mixing DNA template and water for a target volume of 3 µL with 20 ng of DNA and subsequently adding 2 µL of BLT/TB1. The reaction was incubated in a thermocycler according to the manufacturer’s protocol. The reaction was stopped using 1 µL of tagmentation stop buffer dispensed with the I.DOT One and the libraries were washed twice manually with 10 µL of tagmentation wash buffer (TWB). After removal of TWB, 2 µL of NFW, 2 µL of enhanced PCR mix was added with the I.DOT One and 1 µL of IDT® Illumina UD index was added by hand. Libraries were given seven (≥4.9 ng), eight (2.5-4.9 ng), 10 (0.9-2.5 ng), or 14 (<0.9 ng) cycles based on initial genomic DNA concentration. PCRs were incubated using the BLT-PCR program. PCRs were diluted with 17 µL of NFW before transferring 18 µL of diluted sample to a PCR plate containing 16 µL of sample purification beads (SPB) and 18 µL of NFW. After 5 min of incubation, the beads were allowed to pellet on a magnetic rack before 50 µL of supernatant was transferred to a new PCR-plate with 6 µL of SPB. After another incubation step, the beads were washed twice with 45 µL of 80% ethanol and subsequently eluted in 20 µL of NFW. Library concentrations were quantified using the Qubit 1x HS assay on the Tecan infinite F200 PRO before pooling in equimolar concentrations.^21^

The metagenomic sequences were adapter trimmed, quality filtered, and deduplicated using fastp v.0.23.4^22^ with the settings “—correction,” “—detect_adapter_for_pe,” “—cut_right,” “—cut_right_window_size 4,” “—cut_right_mean_quality 20,” “—average_qual 25,” “—length_required 100,” “—dedup,” and “—dup_calc_accuracy 6.” Metagenomic reads that passed quality control were mapped to the human genome, hg38, using bowtie2 v.2.5.0,^23^ and mapped reads were removed from further analysis using samtools v.1.16 for host DNA removal.^24^ Each sample was sub-sampled without replacement to 2.5 million reads. Samples with less than 2.5 million reads were omitted from further analysis.

### 2.4. Microbial composition profiling and analysis

The microbial composition profile of the samples that passed quality control and minimum read requirement was determined using MetaPhlAn4 v.4.1.0,^25^ with the marker gene database mpa_vJun23_CHOCOPhlAnSGB_202307.

The short reads were used for initial microbial composition of the study participants. The analyses were performed in R with the packages, tidyverse,^26^ vegan,^27^ and ampvis2.^28^ Alpha diversity was assessed based on species richness and the Shannon diversity index. Significantly enriched or depleted taxa of the study groups compared to HealthyPouch were evaluated using MaAsLin2 with relative species abundances being centered log ratio normalized.^29^ Beta-diversity was assessed using redundancy analysis (RDA) on Hellinger-transformed relative abundances.

### 2.5. Oxford Nanopore DNA sequencing

Long-read Nanopore sequencing was performed for all SickPouch, HealthyPouch, and NormalGut samples. Prior to library preparation, the DNA was size selected by mixing the sample volume in a 1:1 ratio with AMPure XP beads, and incubated for 10 min on a Hula mixer. After incubation, the beads were washed twice with Long Fragment Buffer (part of the Native Barcoding Kit V14 from Nanopore), air-dried for 30 s, and resuspended in nuclease-free water. Library preparation, barcoding, and flow cell priming were performed using the Native barcoding Kit 24 V14 (SQK-NBD114-24) (Oxford Nanopore Technologies), following the manufacturer’s protocol. Six samples per flowcell were loaded for the HealthyPouch and NormalGut groups, compared to 18 for the SickPouch group. Sequencing was performed on the Oxford Nanopore PromethION 24 (Oxford Nanopore Technologies), equipped with Nanopore R10.4.1 flow cells, at a rate of 400 bases/s, utilizing a sampling rate of 4 or 5 kHz, with subsequent basecalling performed using super accuracy. For samples run with 5 kHz sampling rate, 6 mA and 5 mC DNA methylation was basecalled using the dna_r10.4.1_e8.2_400bps_sup@v4.2.0 v2 and the resultant basecalling file was converted from a BAM to a fastq file using SAMtools v.1.17,^24^ and then demultiplexed into the different barcodes using Dorado v.0.4.1 (https://github.com/nanoporetech/dorado). Reads were trimmed and filtered using Chopper v.0.5.0^30^ to remove sequences shorter than 200 bp or with a Phred quality score below 10. For samples run with 4-kHz sampling rate, trimming and filtering were performed by the MinKNOW software v.22 with Guppy v.6.4 using the same parameters. Metagenomic sequences that passed the quality control were mapped to the human genome, hg38, using minimap2 v.2.24 r1122,^31^ for host DNA removal.

For 5-kHz methylation data, reads were mapped to the assembly disallowing secondary mappings and a methylbed file was generated using the pileup command in modkit v.0.3 (http://github.com/nanoporetech/modkit).

### 2.6. Metagenome-assembled genome recovery

The filtered nanopore reads were assembled and binned using mmlong2-lite v.1.1.0^32^ with the settings “—semibin_model human_gut.” The following tools were used in mmlong2-lite: metaFlye v.2.9.4,^33^ tiara v.1.0.3,^34^ SemiBin2 v.2.1.0,^35^ MetaBat2 v.2.15,^36^ VAMB v.5.0.3,^37^ COMEBin v.1.0.4,^38^ DAStool v.1.1.3,^39^ and CheckM2 v.1.0.2.^40^ GTDB-tk v.2.4.1^41^ using the R226 database^42^ with the setting “—skip_ani_screen” was used for taxonomic classification of the metagenome-assembled genomes (MAGs). The rRNA operon was detected using bakta v.1.9.2^43^ and barrnap v.0.9 (https://github.com/tseemann/barrnap), whereas tRNAs were detected using trnascan-se v.2.0.12.^44^

### 2.7. Plasmid identification and host association

Circular contigs were identified using circure (https://github.com/bertils/circure) and filtered for at least 10× coverage. geNomad v.1.8.1,^45^ plasmer v.23.04.20,^46^ PlasmidHunter v.1.4,^47^ and viralVerify v.1.1^48^ were then used to identify plasmids among the circular contigs, with at least one tool required to classify a contig as a plasmid. To reduce potential false positives, VirSorter2 v.2.2.4^49^ was used to identify viral sequences. For contigs flagged both as plasmid by a plasmid prediction tool and as viral by VirSorter2, geNomad’s plasmid score was used to guide the final classification. Contigs that geNomad did not classify as either plasmid or viral were considered false positives and excluded from further analysis. The identified plasmids were associated with a host based on methylation patterns, using Nanomotif v.5.6^50^ or through the original binning method mmlong2-lite.^32^ If relevant, plasmid replicon types were identified with PlasmidFinder (web-interface),^51^ and gene-level annotation of mobile genetic elements was performed using the MobileOG database v.1.6^52^ with their utility scripts *mobileOGs-pl-kyanite.sh* and *mobileOGs-pl-kyanite.py.*

### 2.8. Identification of antibiotic resistance genes

Antibiotic resistance genes were identified in the contigs ­constructed from the long read sequencing. To do so, RGI main v.6.0.3^53^ with the CARD database v.3.3.0 was employed using the settings “-a DIAMOND,” “—low_quality,” and “—include_nudge.” Only resistance genes identified with the protein homolog model and protein variant model were retained, and filtered for a minimum identity of 80%, and a minimum reference coverage of 80% (as previously used by Lee et al.^54^). All antibiotic resistance genes (ARGs) annotated to confer resistance through antibiotic efflux were removed to lower the number of potential false positives. The presence of *gyrA* and *parC* genes in *E. coli* MAGs was assessed by predicting protein-coding genes using Prodigal v.2.6.3,^55^ followed by DIAMOND v.2.1.8^56^ blastp searches against a reference database of *gyrA* and *parC* sequences retrieved from NCBI RefSeq.

### 2.9. Ethics

The study was performed adhering to the requirements of Good Clinical Practice and the Revised Declaration of Helsinki. All participants provided signed written informed consent to participate. Consent for participation could be withdrawn at any time during the study period. The Regional Research Ethics Committee of Northern Jutland, Denmark, approved the study (project numbers N-20150021 and N-20180013).

## 3. Results

The study comprises four distinct cohorts with a total of 190 participants: individuals with a normally functioning IPAA (HealthyPouch, *n* = 38); patients with chronic pouchitis (SickPouch, *n* = 37), including 11 individuals with antibiotic-dependent pouchitis and 26 with antibiotic-refractory pouchitis; individuals with ulcerative colitis (UC, *n* = 23); and healthy controls with normal gut physiology (NormalGut, *n* = 92). An overview of the study demographics and clinical characteristics is provided in Table 1.

**Table 1.** Clinical and demographic data of the study participants; an overview of the study demographics and statistics.

	SickPouch	HealthyPouch	UC	NormalGut
**Participants, *n***	37	38	23	92
**Age, years, mean (range)**	49 (23-72)	55 (28-76)	50 (24-86)	44.44 (23-72)
**Sex, female/male**	24/13	13/25	12/11	46/46
**Stool frequency per day, median, (range)**	12 (8-25)^a^	5 (3-8)	2 (1-5)	1 (0-3)^b^
**cPDAI mean, (range)**	3.8 (3-6)	1.1 (0-2)	–	–
**SCCAI mean, (range)**	–	–	1.2 (0-4)	–

a Calculated based on complete data from 31 participants.

b Calculated based on complete data from 72 participants.

Abbreviations: cPADI, Clinical Pouchitis Activity Disease Index; SCCAI, Simple Clinical Colitis Activity Index.

The short-read metagenomic data were used to assess the gut microbial composition of the participants. Samples with insufficient read depth were omitted from further analysis (SickPouch: 3/37, HealthyPouch: 1/38, UC: 6/23, NormalGut: 22/92).

The richness and Shannon diversity differ significantly between the colonic microbiomes, UC and NormalGut, and the pouch microbiomes, SickPouch and HealthyPouch (Wilcoxon rank sum test; Bonferroni adjusted *P* < .0001) (Figure 1A, B). Microbial richness varied significantly between the study cohorts being lowest in SickPouch participants and highest in the NormalGut participants (Wilcoxon rank sum test; Bonferroni adjusted *P* < .01). When comparing Shannon diversity, no significant difference in diversity was found between SickPouch and HealthyPouch whereas UC and NormalGut differed significantly (Wilcoxon rank sum test; Bonferroni adjusted *P* < .05).

**Figure 1.**
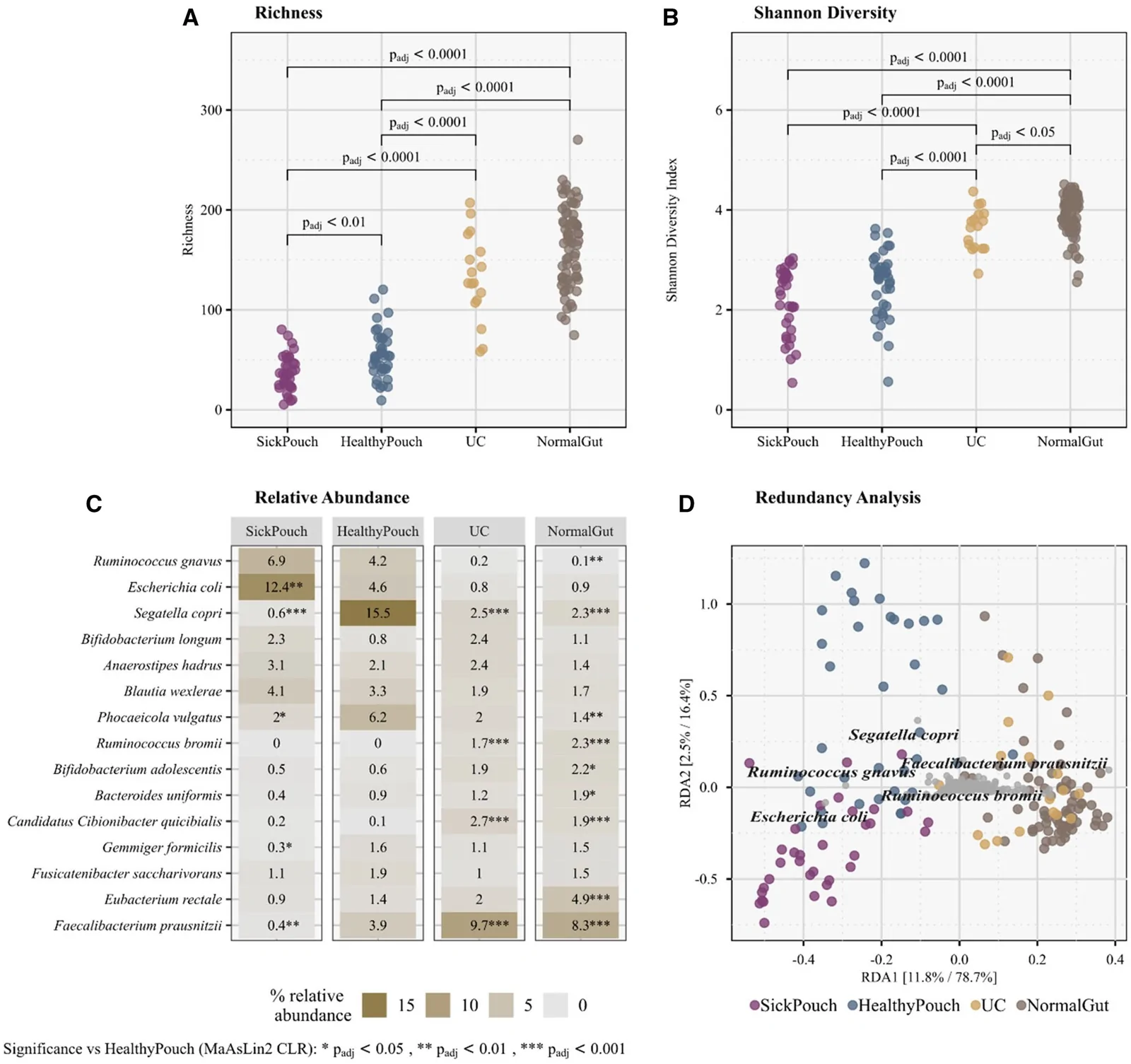
Overview of the microbial composition profile for the study cohorts. (A) Species richness calculated as the number of species with a relative abundance above zero. (B) Shannon diversity index. Points represent fecal samples colored according to the study group. *P*-values comparing study groups were calculated using a Wilcoxon rank sum test. Bonferroni was used to adjust *P*-values for multiple hypothesis testing. *P*-values are only illustrated if the difference between groups was statistically significant. (C) Heatmaps showing the mean relative abundances of the 15 most abundant species across the SickPouch, HealthyPouch, UC, and NormalGut groups. Significant species relative abundance differences between study groups vs HealthyPouch indicated by Benjamini–Hochberg adjusted *P*-values (*P*_adj_). (D) Redundancy analysis (RDA) of Hellinger-transformed relative abundances, constrained by the study group. The points are colored according to the study group. The first eigenvalue of the axis labels signifies the axis’ contribution to the total inertia within the data. The second eigenvalue of the axis labels indicates the axis’ contribution only within the constrained space inertia. In total, the study groups explain 14.3% of the total inertia in the data, as indicated by the cumulative eigenvalue. The gray points represent species contributions to the axes and genera of the five most contributing species are labeled. CLR, centered log ratio.

The SickPouch microbiome was characterized by a high relative abundance of *Escherichia coli* and *Ruminococcus gnavus* (Figure 1C), whereas the HealthyPouch microbiome had a higher relative abundance of *Segatella copri*, *Phocaeicola ­vulgatus*, and *Faecalibacterium prausnitzii*. *F. prausnitzii* had the highest average relative abundance in both the UC and NormalGut group. *S. copri* (MaAsLin2 CLR, Benjamini-Hochberg [BH] adjusted *P* < .001) and *Phocaeicola vulgatus* (MaAsLin2 CLR, BH adjusted *P* < .05) were significantly enriched in the HealthyPouch group compared to SickPouch, whereas *Faecalimonas umbilicate* (MaAsLin2 CLR, BH adjusted *P* < .001) and *E. coli* (MaAsLin2 CLR, BH adjusted *P* < .01) were significantly depleted. The HealthyPouch microbiome was significantly depleted of *F. prausnitzii* (MaAsLin2 CLR, BH adjusted *P* < .001) as well as several other taxa compared to both UC and NormalGut, whereas *S. copri* was significantly enriched (MaAsLin2 CLR, BH adjusted *P* < .001). To identify the relationship between the study groups and the relative abundance of species present in the samples, an RDA with the study group as the constrained variable was performed using Hellinger-transformed relative abundances (Figure 1D). The UC group and NormalGut microbiome form a joint cluster that is distinct from both the SickPouch and HealthyPouch clusters. The grouping patterns were also observable in unconstrained ordination, principal components analysis (PCA) (Figure S1). The SickPouch group forms a distinct cluster mainly driven by the species *E. coli* and *R. gnavus*. The HealthyPouch group is more divided with some microbiomes more similar to the SickPouch group and others forming a distinct HealthyPouch subgroup. This distinctive subgroup is driven primarily by an increase in relative abundance of *S. copri*. The joint cluster formed by the NormalGut and UC samples were driven predominantly by the relative abundance of the species *F. prausnitzii* and *R. bromii*. RDA confirmed that health status significantly explains microbiome com­position (*F* = 9.03, *P* < .0001, 9999 permutations). Pairwise PERMANOVA showed significant differences between all group pairs (all BH adjusted *P* ≤ .003, 999 permutations), with the strongest separation between SickPouch and NormalGut (*F* = 18.1, R2 = 0.15). A betadisper test revealed significant heterogeneity of dispersions (*P* = .001), driven by differences between SickPouch and NormalGut (Tukey adjusted *P* < .0001), NormalGut and HealthyPouch (Tukey adjusted *P* < .0001), and SickPouch and UC (Tukey adjusted *P* < .001), indicating PERMANOVA results for these pairs reflect both compositional differences and unequal within-group variability.

After the discovery of distinct microbial communities of the groups from the short-read dataset, we investigated the effects of prolonged exposure to antibiotics, most commonly ciprofloxacin and metronidazole, in the SickPouch group. ARGs are frequently located on mobile genetic elements such as plasmids and genomic islands, which are critical for horizontal gene transfer between bacteria. Hence, to properly investigate the effect of antimicrobial resistance and associate plasmids to their host genomes, it is essential to recover complete, contiguous MAGs. We therefore created the hitherto largest human fecal long-read dataset totaling 3766 Gb from 238 fecal samples (Figure S2A). Of these 156 samples were used for MAG recovery, from which 5999 high-quality (228 circular) and 6283 medium-quality MAGs were recovered. Table S1 presents an overview of sequencing and assembly statistics as well as MAGs recovered in each group.

Of the 12 282 MAGs, only 213 lacked species-level assignment by GTDB. The low taxonomic novelty supports the validity of the short-read analysis as the majority of the taxa are well represented in the existing reference databases at the species level (Figure S2C). However, comparing the HQ MAGs to the reference genomes in GTDB (excluding type strains), the study MAGs are about 10 times more contiguous (median N50: 49 kb [IQR: 73.9 kb] vs 459 kb [IQR: 1000 kb]) (Figure S2B). The 5999 HQ MAGs recovered in the study were assigned to 884 different species spanning 398 genera using GTDB R226. Of these, 60 genera and 262 species clusters did not have a full-length 16S rRNA gene (SSU) associated in GTDB, but will gain one or more with the addition of the study MAGs. This is essential to enable linkage of the thousands of 16S rRNA gene amplicon studies to genomic information.

The most common fluoroquinolone resistance mechanisms that confer the highest levels of antibiotic resistance are chromosomal single nucleotide polymorphism (SNP) mutations in the antibiotic target genes encoding DNA gyrase, *gyrAB*, and topoisomerase IV, *parCDE*.^57^^,^^58^ Due to the high prevalence of *E. coli* in the SickPouch cohort, the analysis focused specifically on the *E. coli* MAGs. For each gene, only MAGs with the gene identified were included in the analysis. In the SickPouch group, 5/21 recovered *E. coli* MAGs had a *gyrA* resistance allele, which was significantly higher than in the NormalGut group (Fisher’s exact test; BH adjusted *P* < .05). Additionally, 6/12 recovered *E. coli* MAGs from the SickPouch group had a *parC* resistance allele, which was significantly higher than both the HealthyPouch (Fisher’s exact test; BH adjusted *P* < .05) and NormalGut (Fisher’s exact test; BH adjusted *P* < .05). No statistically significant difference could be observed between the HealthyPouch and NormalGut *E. coli* for either of the resistance alleles (Table 2).

**Table 2.** *Escherichia coli* MAGs with *gyrA/parC* mutations for study groups; number of *E. coli* MAGs with identified *gyrA/parC* mutations.

	SickPouch	HealthyPouch	NormalGut	OR (CI) *P*-value (SickPouch ∼ HealthyPouch)	OR (CI) *P*-value (SickPouch ∼ NormalGut)	OR (CI) *P*-value (HealthyPouch ∼ NormalGut)
***E. coli* bins, *n***	21	26	42^a^	–	–	–
***n* (%) of MAGs with *gyrA* resistance allele**	5 (23.8)	1 (3.8)	1 (2.4)	7.5	12	1.6
				(0.7-383)	(1.2-603)	(0.0-129)
				*P*_adj_ = .114	*P*_adj_ = .028	*P*_adj_ = 1.000
***n* (%) of MAGs with *parC* resistance allele**	6 (28.6)	0 (0)	2 (4.9)	22.2^b^	7.5	0.3^b^
				(1.7-Inf)	(1.2-84.3)	(0.0-8)
				*P*_adj_ = .028	*P*_adj_ = .028	*P*_adj_ = .666
***n* (%) of MAGs with co-occurrence**	5 (23.8)	0 (0)	1 (2.4)	17.7^b^	11.7	0.5^b^
				(1.3-Inf)	(1.2-589)	(0-60)
				*P*_adj_ = .028	*P*_adj_ = .028	*P*_adj_ = 1.000

Fisher’s exact test was used to test for significant enrichment of *gyrA/parC* mutations in each group. Benjamini–Hochberg was used to adjust *P*-values for multiple hypothesis testing.

a One *E. coli* MAG from the NormalGut group lacked *gyrA* and one lacked *parC*; both were excluded from the respective statistical tests.

b Odds ratio calculated using Haldane–Anscombe continuity correction (0.5 added to all cells) due to zero events in the comparison group.

Abbreviations: OR, odds ratio; CI, 95% confidence interval; MAG, metagenome-assembled genome.

To complement the chromosomal ARG analysis and determine the host context of ARG-carrying plasmids, a methylation-based plasmid to MAG linkage approach was applied to assign plasmids to their bacterial hosts. In the SickPouch group, 18 plasmids were associated with an *E. coli* MAG by matching DNA methylation patterns using Nanomotif,^50^ contributing approximately one plasmid per MAG that would not have been linked using the original binning approach without DNA methylation. Of the putative plasmids associated with an *E. coli* MAG in the SickPouch group, three carried one or more ARGs. All three plasmids encoded extended-spectrum beta-lactamase (ESBL) genes, belonging to either the TEM or SHV families (Figure 2A, Figures S3 and S4). For example, one plasmid classified as an IncI1-I(Alpha) replicon type and carrying the ESBL gene *SHV-12* was associated with its corresponding MAG based on matching methylation patterns. In addition to the ARG, the plasmid carried multiple genes associated with the conjugation machinery, including *mob*, *tra*, and *trb* genes along with additional genes involved in replication such as the *repA* gene, as well as genes involved in initiation, integration/excision, recombination, repair, stability, transfer, and defense (Figure 2A). The plasmid is associated with the *E. coli* MAG through comparison of methylation patterns of the bins present in the sample as well as the plasmid of interest. The *E. coli* MAG and the putative plasmid share identical methylation levels on several methylation motifs, not present in other recovered MAGs, specifically on the motifs TCC**_6m_A**(N)_7_TGC and G**_6m_A**TC (Figure 2B).

**Figure 2.**
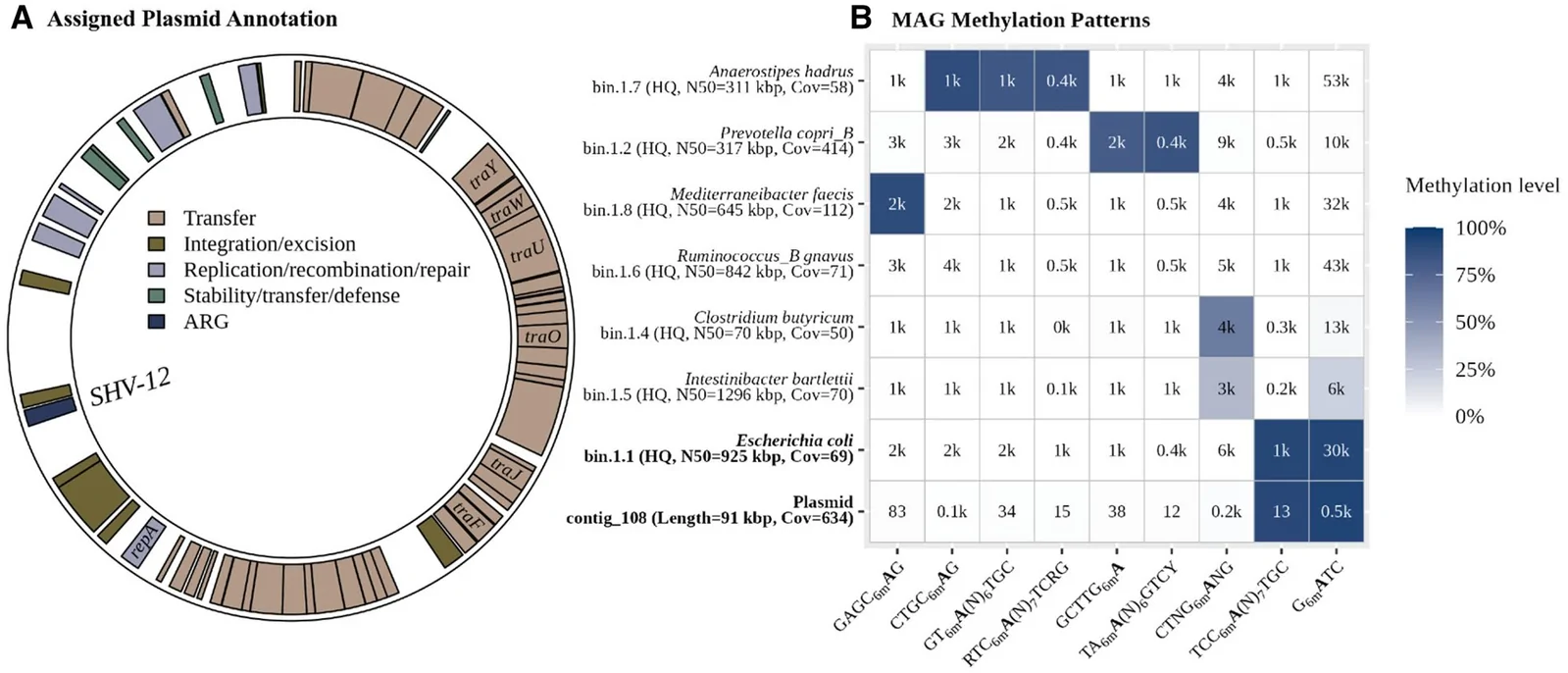
Plasmid characteristics and host association. (A) Plasmid with gene-level annotation of common plasmidial genes. The genes are colored according to gene function, and annotated with gene names where relevant. The identified plasmid is contig 108 from patient sample PaPr00000245MP. (B) Heatmap showing the methylation levels of bins in the sample and specific plasmid. The number inside the tiles denotes the number of motif occurrences in the MAG or plasmid. *X*-axis: subset of methylated motifs identified by Nanomotif. *Y*-axis: the plasmid and HQ bins recovered in the sample. The mean read methylation for each motif (with ≥5 motif observations) is calculated for motif observations with at least 10 read mappings. The median value of these is then reported as the motif methylation level.

## 4. Discussion

The primary treatment for chronic pouchitis is antibiotic therapy. While FMT has proven effective for recurrent *C. difficile* infections and preliminary studies suggest potential benefit in UC, its efficacy in treating chronic pouchitis remains limited.^13–15^^,^^59^^,^^60^ This study, therefore, aimed to characterize the defining features of a “healthy” ileal pouch microbiome, placing these features in the broader context of chronic pouchitis, UC, and normal colonic physiology. This study demonstrates that the HealthyPouch microbiome represents a distinct microbial composition, differing significantly from both NormalGut and UC. The findings highlight the pouch as a unique microbial niche potentially shaped by disease state and antibiotic exposure.

The microbial profile of the SickPouch, Healthypouch, UC, and NormalGut are largely concordant with findings in Reshef et al.^61^ and Dubinsky et al.,^62^ with the healthy group with normal gut physiology showing significantly increased alpha diversity compared to the pouch (inflamed and non-inflamed) microbiomes. In our study, the HealthyPouch alpha diversity metrics, richness and Shannon diversity, were significantly lower than for the UC microbiome (BH adjusted *P* < .0001), which was not the case in Reshef et al.^61^ or Dubinsky et al.^62^ This difference may reflect differences in the cohorts. As reported previously, *E. coli* is significantly enriched in chronic pouchitis cases compared to healthy non-IBD controls, whereas *F. praunitzii* and *R. bromii* were significantly depleted in both the chronic pouchitis and non-inflamed pouches.^61^^,^^62^ While Dubinsky et al.^62^ found *S. copri* to be enriched, though not significantly so, in UC and normal pouch compared to pouchitis, our study found a significant enrichment specific to HealthyPouch across all comparisons (MaAsLin2 CLR, BH adjusted *P* < .001), suggesting a potential important role in pouch health that warrants further investigation. *S. copri* (previously *Prevotella copri*) is an obligately anaerobic, gram-negative bacterium^63^ often found in high relative abundance in non-western fiber-rich diets.^64^ The observed difference may reflect differences in cohort intake of dietary fibers. It should be noted that antibiotic use in the SickPouch group represents a confounding factor in interpreting the observed microbial differences. Stool frequency and consistency are known to be major drivers for alterations in the gut microbiome diversity.^65^^,^^66^ In fact, Turpin et al.^67^ reported that endoscopically inactive patients showed similar differences in overall microbial composition between low and high stool frequency to patients with active inflammation, suggesting that symptoms may persist even in the absence of clear inflammation and correlate with differences in the mucosa-associated microbiota. In our cohort, stool frequency differs between HealthyPouch and SickPouch patients and is higher in both pouch groups than in NormalGut and UC participants (Table 1), indicating that stool frequency may contribute to the observed group differences.

Short-read sequencing often produces incomplete, highly fragmented MAGs, which is a severe limitation in genomic studies. We therefore created the largest fecal long-read dataset recovering more than 12 000 MAGs, of which 5999 are of HQ. Despite the human fecal microbiome being one of the most extensively studied microbial systems, with previous efforts recovering more than 150 000 MAGs (70 000 HQ, N50 42 kb, median number of contigs 104) representing almost 5000 species, significant gaps remain in genome quality of the reference databases.^68^ Compared to GTDB R226, our MAGs are about nine times more contiguous than the non-type strain GTDB representative genomes to which they were mapped. Furthermore, 29% of our HQ MAGs with a near full-length SSU gene were assigned to GTDB species clusters that lack such a gene (15% for genus clusters), highlighting a significant gap in SSU representation. Integrating our HQ, contiguous MAGs into a future release of GTDB will substantially improve reference genome quality and expand underrepresented clusters. Additionally, the higher contiguity and better representation of SSUs underscore the importance of generating long-read MAGs for proper analysis of difficult-to-assemble genomic regions, which are often found in mobile genetic elements.^69^^,^^70^

The SickPouch group shows elevated levels of *E. coli*, whose characteristics—such as rapid doubling time suited to the high stool frequency of the pouch, the ability to persist in the mucosa, and benefit from inflammation—may enhance adaptability and contribute to disease severity.^71^ Consistent with this, Dubinsky et al.^72^ demonstrated, using an inference framework for bacterial growth (iREP), that *E. coli* from patients with a pouch had a higher growth rate compared to *E. coli* in non-IBD microbiomes. Additionally, we find that *E. coli* exhibits elevated occurrence of specific ARGs in patients with pouchitis. The first-line treatment for pouchitis, especially in chronic cases, typically involves repeated or prolonged use of metronidazole and ciprofloxacin.^6^ This regimen has been associated with an increased occurrence of ARGs in the pouch microbiome.^72^^,^^73^ While previous work has examined these patterns using short-read assembled MAGs, our approach leverages long read assemblies to provide improved genomic continuity and resolution of these elements within their broader genomic context. Using our extensive MAG catalog from HealthyGut, NormalGut, and SickPouch, we show this increased susceptibility for ciprofloxacin at individual *E. coli* MAG level for these groups. *E. coli* in the SickPouch microbiomes have a significantly increased occurrence of SNP mutations in the *parC* genes and co-occurence of *gyrA* and *parC* resistance alleles (Table 2), the most common ciprofloxacin resistance mechanism, compared to *E. coli in* the other study groups.

In our case, three patients with chronic pouchitis were found to harbor *E. coli* carrying a plasmid with an ESBL gene. Though the ARGs found on the plasmids do not confer resistance to the reported consumed antibiotics, these plasmids are still a major clinical concern. Additionally, multiple genes involved in the conjugation machinery were identified on the same plasmids, indicating that these ARG-carrying plasmids are capable of transferring between bacteria. These characteristics make these plasmids of clinical interest, potentially rendering future antibiotic treatment ineffective for these specific patients. Although *E. coli* is well known to carry conjugative plasmids encoding ESBL genes, this highlights the importance of accurately linking plasmids to their microbial hosts in metagenomic analyses, while also underscoring the capacity of the SickPouch microbiome to harbor and potentially disseminate antibiotic resistance elements.^74^^,^^75^ Without such associations, resistance genes carried on plasmids may be overlooked, limiting our understanding of their clinical relevance and pathogen treatment options. One way to improve patient response to antibiotic therapy is through personalized treatment based on the resistance profile of their gut microbiome. For instance, a patient with high *E. coli* abundance carrying ciprofloxacin resistance genes may benefit more from alternative antibiotic treatments. In this context, antibiotic-refractory chronic pouchitis may reflect a resistance phenomenon, suggesting that the microbial community members involved have developed mechanisms to evade standard antibiotic therapies, thereby contributing to persistent inflammation despite treatment. Several studies have shown that FMT can, in some cases, replace conspecific strains, highlighting its potential as an alternative to antibiotic therapy.^76^^,^^77^ As metagenomics become more integrated into clinical practice, accessible tools for plasmid–host association and strain-level resolution will be crucial for advancing microbiome-informed therapy and responsible antibiotic use.

From a therapeutic perspective, the distinct composition of the HealthyPouch microbiota—closer to a balanced state yet different from NormalGut—suggests that it could serve as a more suitable donor community for FMT in chronic pouchitis treatment. Supporting this hypothesis, we have previously conducted a proof of concept FMT study in which three out of three chronic pouchitis patients achieved clinical remission after a 1-month treatment period with FMT from a single HealthyPouch donor.^78^ However, larger randomized control studies are needed to confirm these preliminary findings. Additionally, our results emphasize that recurrent antibiotic exposure not only reduces microbial diversity but also drives resistance evolution in key taxa such as *E. coli.* Future research should focus on personalized approaches to antibiotic stewardship, the development of microbiome-based therapies tailored to pouch-specific ecology, and continuous monitoring of resistance genes and plasmid transmission within this unique microbiome.

## Supplementary Material

jjag150_Supplementary_Data

## Data Availability

This study uses a subset of all DNA sequencing data generated. All DNA sequences have been deposited at the European Nucleotide Archive under the accession number PRJEB98069. Additionally, we incorporated data from previous studies, which are publicly available under accession numbers PRJEB80556 and PRJEB66493. Source code for recreating plots can be found at: https://github.com/SebastianDall/HealthyPouch_article.
