# Supplementary material for: Microbial and antimicrobial resistance signatures differentiate healthy and inflamed ileal pouches in patients with ulcerative colitis
Source: J Crohns Colitis. 2026 Nov 7;20(11):jjag150. doi: 10.1093/ecco-jcc/jjag150 (PMC13744404; doi:10.1093/ecco-jcc/jjag150)
Supplement: Supplementary file 1 [file jjag150_supplementary_data.zip › ecco-jcc-2026-0370-37-47.pdf]

700 # Supplementary

701 **Supplementary Table 1. Long-read sample statistics.** Sample statistics for sequencing, assembly, and binning for
702 each group. Statistics are reported as median and inter-quartile range are in parenthesis unless otherwise indicated.
703 "Samples w. 5kHz" refers to the number of samples that was sequenced using 5kHz sampling rate on the Oxford
704 Nanopore. "Percentage of assembly length binned" is the sum of the binned contigs" length divided by the assembly
705 length. HQ: high-quality, MQ: medium-quality.

	**SickPouch**	**HealthyPouch**	**NormalGut**
Samples, n	32	36	88
Samples w. 5kHz, n	28	25	54
Sequencing depth [Gbp]	3.19 (2.39)	11.6 (8.37)	22.1 (17.8)
Read N50 [bp]	1742 (1067)	2552 (930)	4022 (1872)
Assembly length [Mbp]	40 (40)	138 (96)	484 (250)
Number of contigs	4368 (4092)	10300 (6184)	23695 (13717)
Assembly N50 [bp]	24877 (43487)	39600 (26515)	63858 (58798)
Percentage of assembly length binned	63% (13%)	62% (8%)	63% (10%)
Total:			
HQ MAGs, n	162	679	5158
MQ MAGs, n	170	616	5497
Specific:			
HQ *E. coli* MAGs, n	20	22	32
MQ *E. coli* MAGs, n	1	4	10

For Review Only

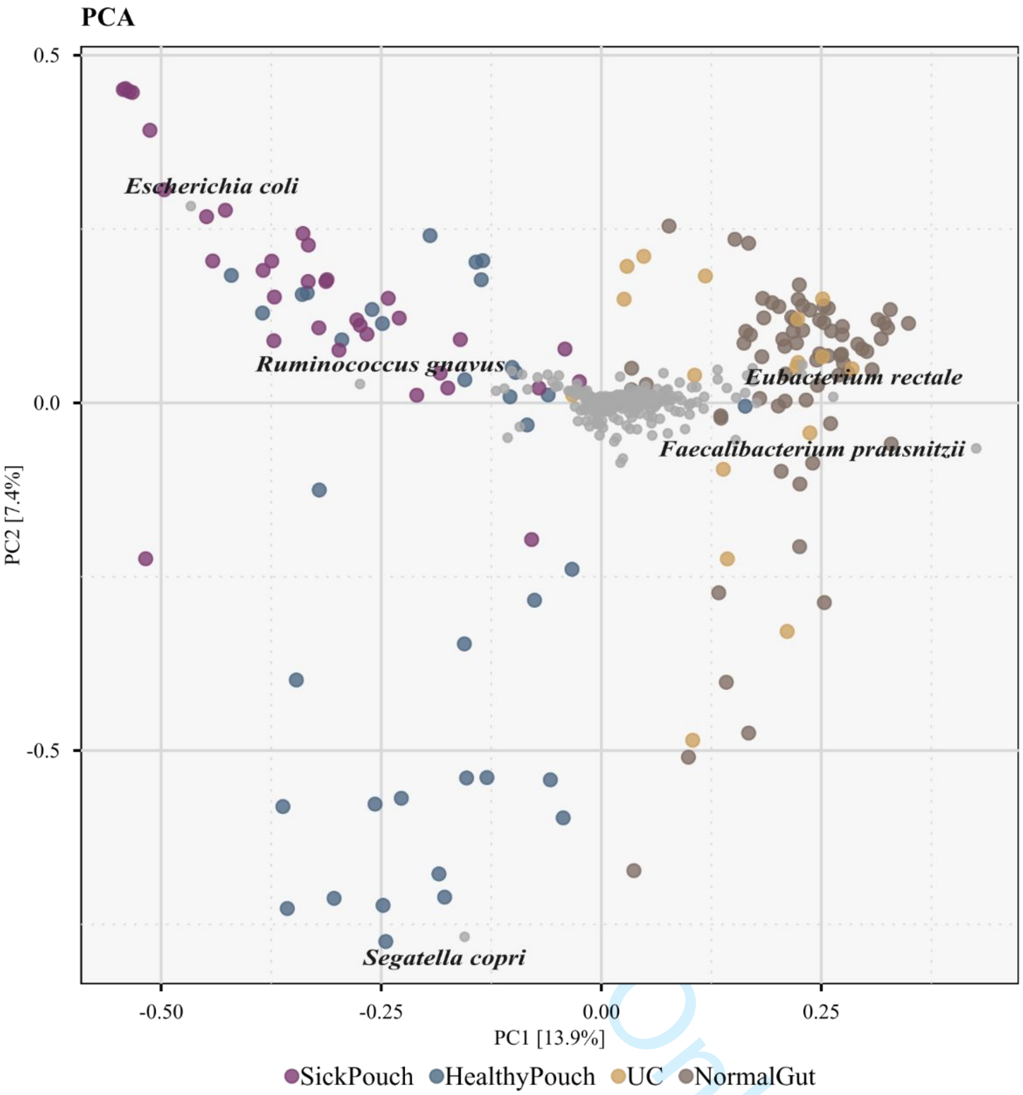

706

707 **Supplementary Figure 1. Principal component analysis.** Principal component analysis (PCA) of Hellinger transformed
708 relative abundances. The points are colored according to the study group. The grey points represent species
709 contributions to the axes and genera of the 5 most contributing species are labelled.

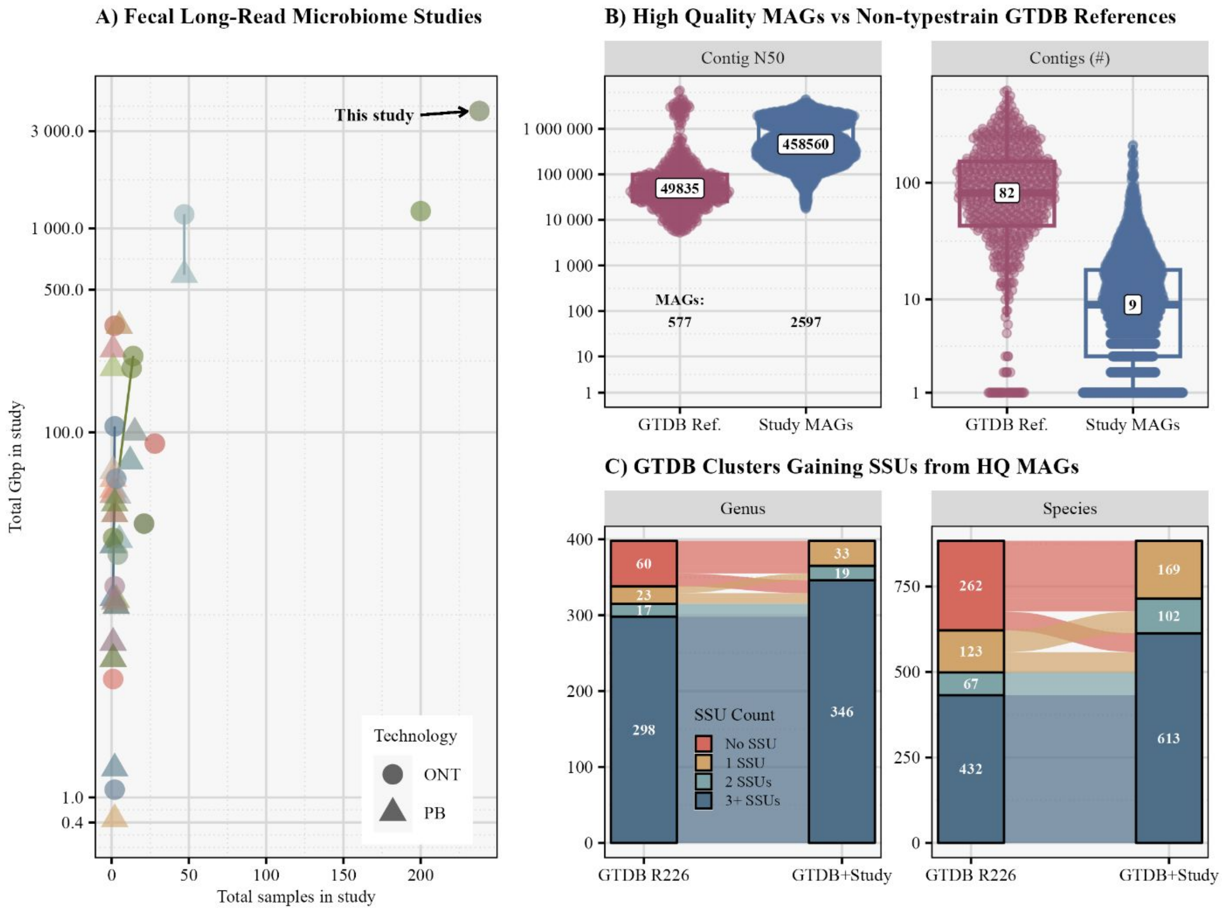

Supplementary Figure 2. Overview of long-read dataset. A) Size of fecal long-read datasets adapted from Minich et al.[79]. B) Contig N50 and number of contigs for GTDB R226 reference genomes that are non-typestrains vs HQ MAGs recovered in this study with the same non-typestrain species taxonomy. C) Number of genera and species clusters with 0, 1, 2 or 3+, 16S rRNA small sub-units (SSUs) that will gain SSUs by the addition of these HQ MAGs to GTDB R226. A SSU was defined as the 16S rRNA gene being at least 1200 bp for Bacteria and at least 900 bp for Archaea.[80] ONT: Oxford Nanopore Technologies, PB: Pacific Biosciences.

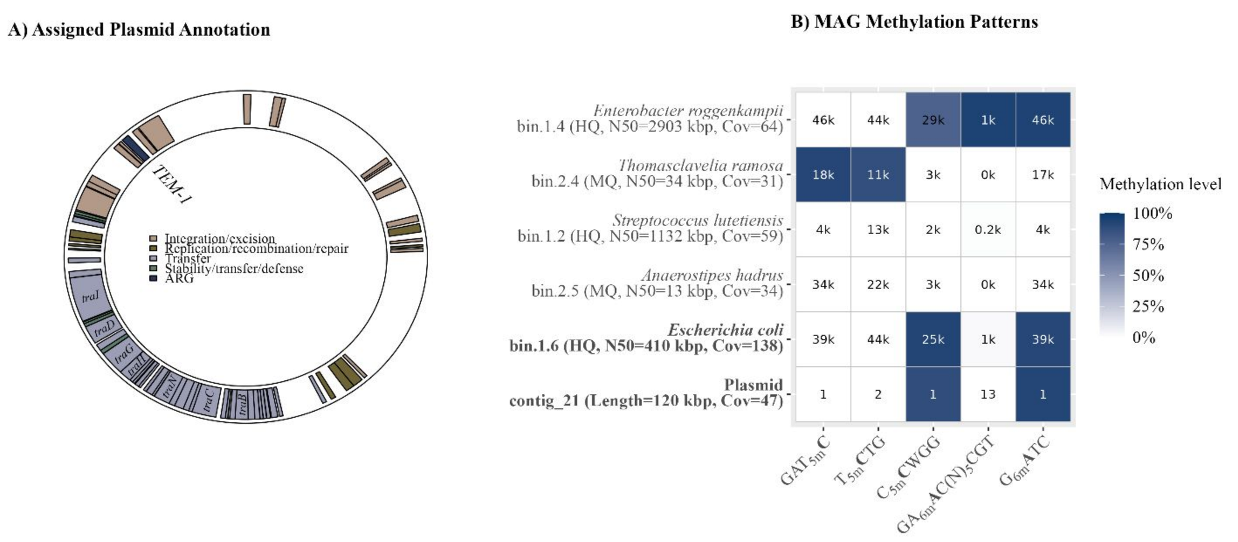

Supplementary Figure 3. Plasmid characteristics. ARG carrying plasmid encoding the ESBL *TEM-1*. Contig 21 from patient sample PaPr00000001MP annotated for plasmidial genes using the mobileOG database and utility scripts. The plasmid replicon type is classified as IncFIB using PlasmidFinder. The plasmid is binned to an *E. coli* MAG based on methylation patterns using Nanomotif. This plasmid would not have been assigned to a MAG using the primary binning method.

For Peer Review Only

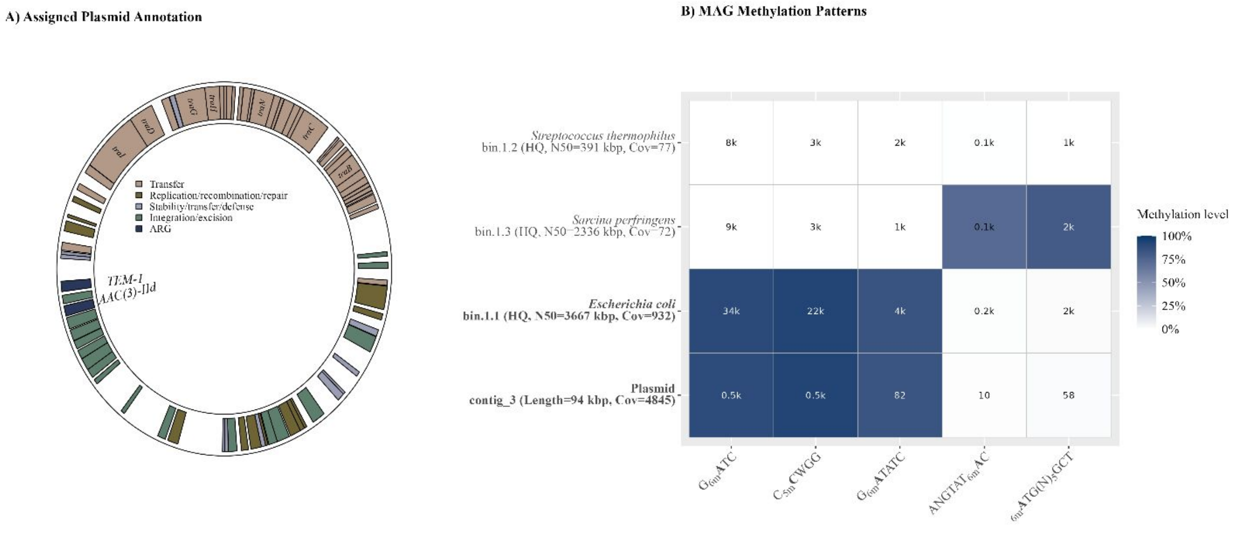

Supplementary Figure 4. Plasmid characteristics. ARG carrying plasmid encoding *AAC(3)-IID* and the ESBL *TEM-1*. Contig 3 from patient sample PaPr00000088MP annotated for plasmidial genes using the mobileOG database and utility scripts. The plasmid replicon type is classified as IncFII using PlasmidFinder. The plasmid is binned to an *E. coli* MAG based on methylation patterns using Nanomotif. This plasmid would not have been assigned to a MAG using the primary binning method.

For Review Only

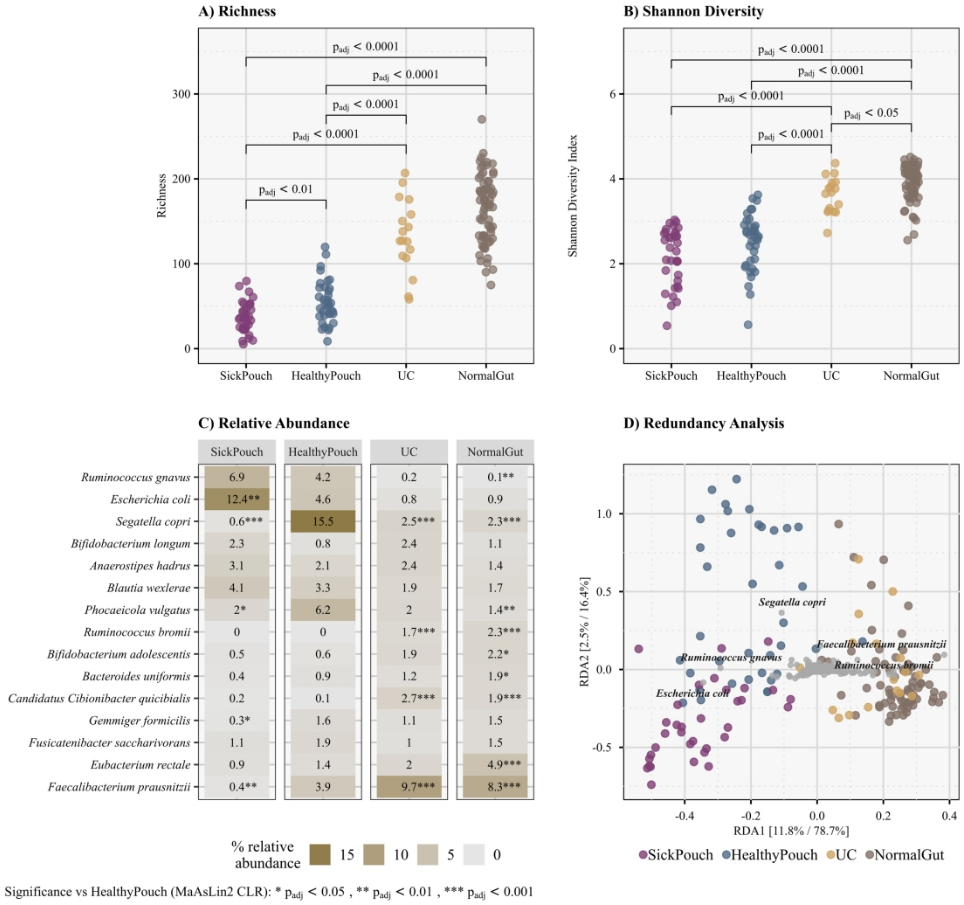

229x229mm (300 x 300 DPI)

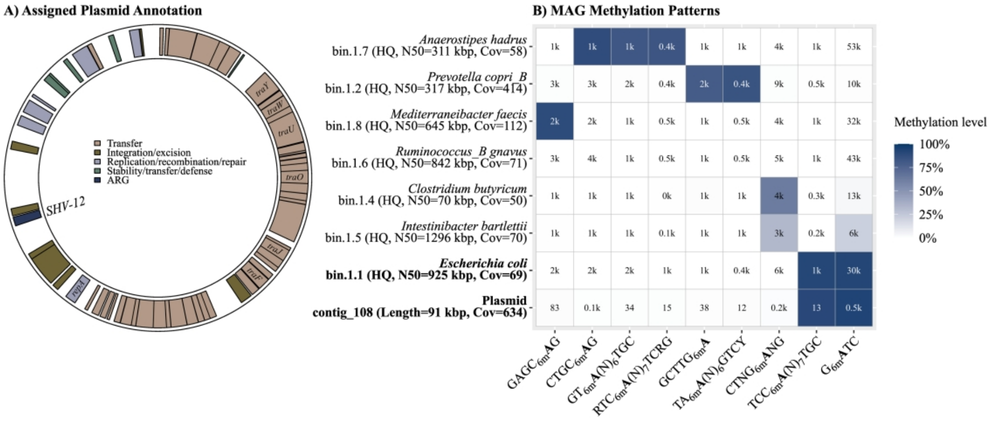

323x138mm (300 x 300 DPI)

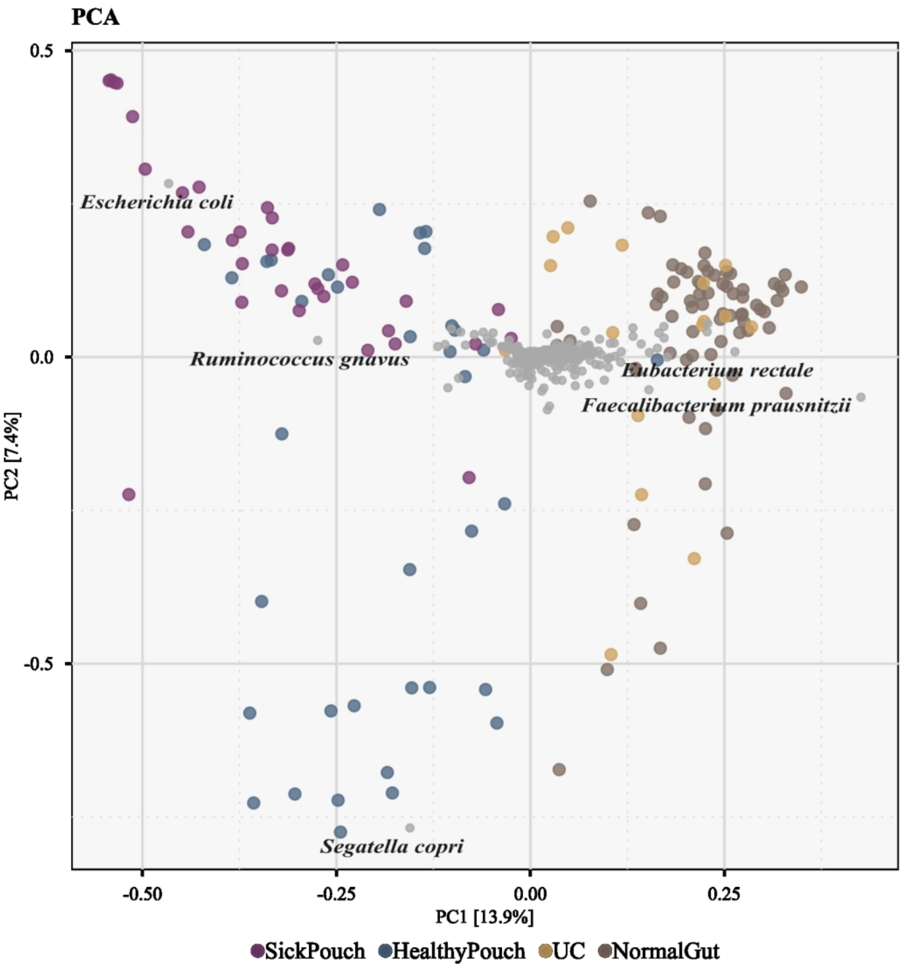

150x150mm (300 x 300 DPI)

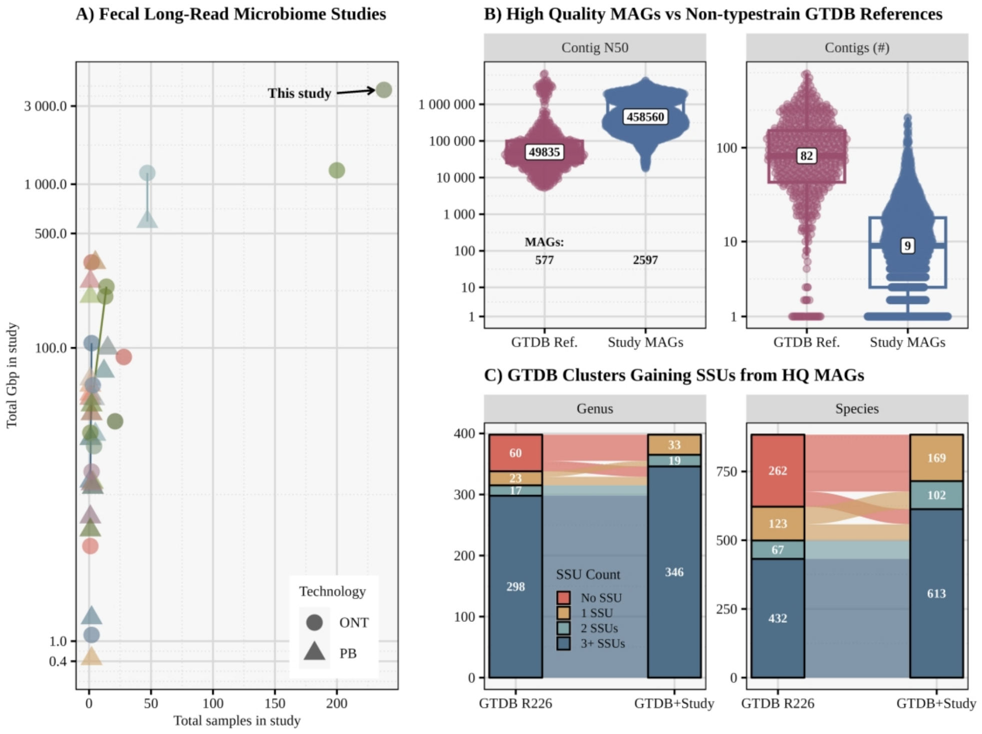

198x150mm (300 x 300 DPI)

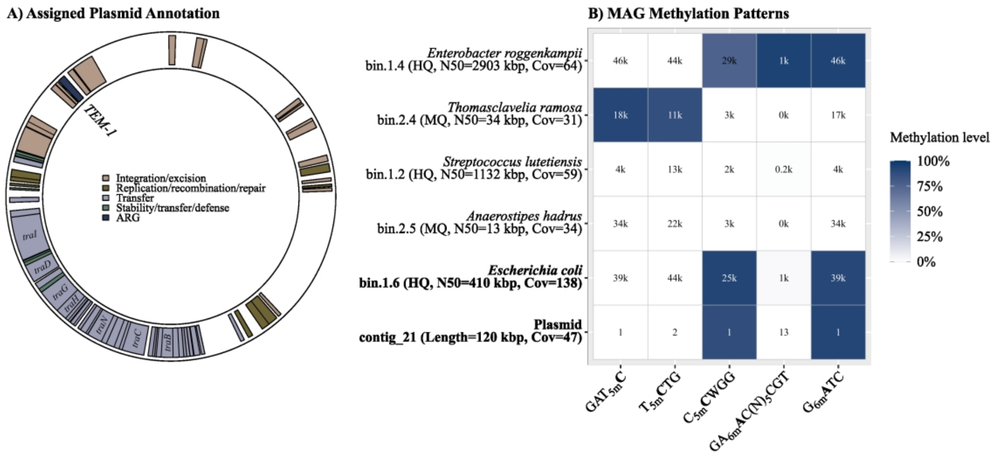

300x139mm (300 x 300 DPI)

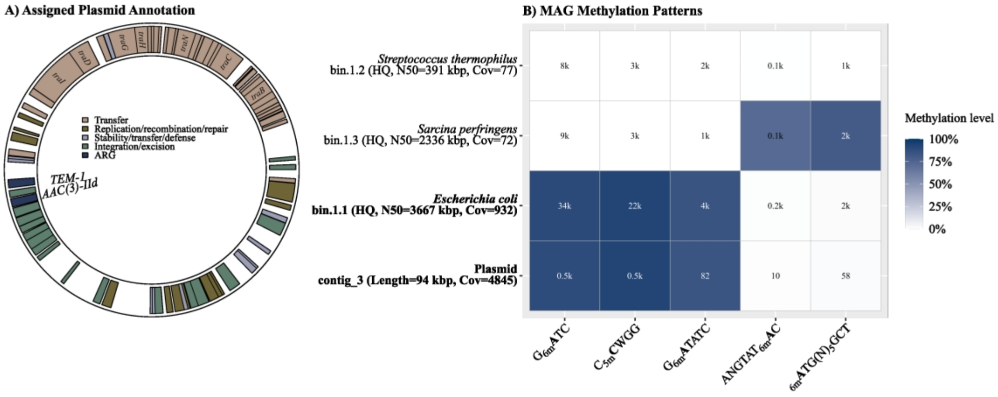

337x134mm (300 x 300 DPI)
